# Ability of individuals with chronic hemiparetic stroke to locate their forearms during single-arm and between-arms tasks

**DOI:** 10.1371/journal.pone.0206518

**Published:** 2018-10-29

**Authors:** Netta Gurari, Justin M. Drogos, Julius P. A. Dewald

**Affiliations:** 1 Department of Physical Therapy and Human Movement Sciences, Northwestern University, Chicago, Illinois, United States of America; 2 Department of Physical Medicine and Rehabilitation, Northwestern University, Evanston, Illinois, United States of America; 3 Department of Biomedical Engineering, Northwestern University, Evanston, Illinois, United States of America; 4 University of Twente, Department of Biomechanical Engineering, Faculty of Engineering Technology, Enschede, The Netherlands; University of Ottawa, CANADA

## Abstract

**Background:**

According to between-arms assessments, more than 50% of individuals with stroke have an impaired position sense. Our previous work, which employed a clinical assessment and slightly differing tasks, indicates that individuals who have a deficit on a between-forearms position-localization task do not necessarily have a deficit on a single-forearm position-localization task.

**Objective:**

Our goal here was to, using robotics tools, determine whether individuals with stroke who have a deficit when matching forearm positions within an arm also have a deficit when mirroring forearm positions between arms, independent of the arm that leads the task.

**Methods:**

Eighteen participants with chronic hemiparetic stroke and nine controls completed a single-arm position-matching experiment and between-arms position-mirroring experiment. For each experiment, the reference forearm (left/right) passively rotated about the elbow joint to a reference target location (flexion/extension), and then the participant actively rotated their same/opposite forearm to match/mirror the reference forearm’s position. Participants with stroke were classified as having a position-matching/-mirroring deficit based on a quantitative threshold that was derived from the controls’ data.

**Results:**

On our single-arm task, one participant with stroke was classified as having a position-matching deficit with a mean magnitude of error greater than 10.7° when referencing their paretic arm. Position-matching ability did not significantly differ for the controls and the remaining seventeen participants with stroke. On our between-arms task, seven participants with stroke were classified as having a position-mirroring deficit with a mean magnitude of error greater than 10.1°. Position-mirroring accuracy was worse for these participants with stroke, when referencing their paretic arm, than the controls.

**Concluding remark:**

Findings underscore the need for assessing within-arm position-matching deficits, in addition to between-arms position-mirroring deficits when referencing each arm, to comprehensively evaluate an individual’s ability to locate their forearm(s).

## Introduction

By 2030 approximately 8.4 million American adults are projected to have survived a stroke [1], and current literature suggests that upwards of 50% of these survivors may have an inability to locate their limb(s) in space [2, 3]. Our original hypothesis for why survivors of a stroke may not accurately locate their limb(s) in space was that they had deficits in locating their paretic limb, and possibly their non-paretic limb, independently. Yet, our first study revealed that an individual with chronic hemiparetic stroke who has a deficit when mirroring their forearms on a clinical between-arms task does not necessarily have a deficit when locating their paretic forearm and non-paretic forearm, separately, on a robotic single-arm position-matching task [4]. Therefore, we now hypothesize that the deficit arose due to the: i) comparison of inaccurate position information relayed from either/both arm(s) and/or ii) inaccurate processing of accurate position information that was relayed from either/both arm(s). Our findings in this earlier study, moreover, showed that while participants with stroke did not significantly differ from controls when matching positions within each arm if they controlled how their forearm moved (i.e., active movements), participants with stroke had a slightly greater variability than controls when matching positions if their forearm was moved for them (i.e., passive movements). Limitations of this earlier work include that a robotic system was used to quantify participant forearm-localization ability on the single-arm task, yet not on the between-arms task. Furthermore, purely passive or purely active movements were employed on the single-arm task, yet a combination of passive and active movements were employed on the between-arms task. We addressed the limitations of our earlier work here by using a robotic system to determine whether individuals with stroke had deficits on a single-arm position-matching task and a between-arms position-mirroring task when a combination of passive and active movements was employed.

As highlighted by Daniel Goble in 2010, the choice of a single-arm task versus a between-arms task is not trivial [5]. Our 2017 article mentioned above underscores this point [4], and the work of Hirayama et al. in 1999 further addresses this notion [6]. Hirayama et al. showed that the ability of patients with lesions to use their non-paretic hand to detect the location of their paretic thumb in space (i.e., thumb localizing test) does not necessarily correlate with their ability to detect the direction of movement passively occurring at their paretic digits (i.e., joint position and movement sense). That is, participants who were impaired when locating their paretic thumb were not necessarily impaired when detecting the direction of movement of their paretic digits, and, vice versa, individuals who were impaired when detecting the direction of movement of their paretic digits were not necessarily impaired when locating their paretic thumb. As indicated in these articles, findings from single-arm and between-arms tasks may lead to differing conclusions.

We briefly cover the neural processes governing single-arm and between-arms position-localization tasks. For each task, numerous afferents (e.g., muscle spindles, joint mechanoreceptors, cutaneous mechanoreceptors) relay sensory information to the cortex via the dorsal columns [7–9]. The sensory information combines with one’s understanding of the size and shape of their body to locate each limb in space [10, 11]. A history-dependent change in the passive mechanical muscle properties, known as muscle thixotropy, can alter the sensory signals during passive movements [12, 13]; yet, these effects can be controlled for by a quick stretch of the muscle [12, 14, 15]. In addition, an efference copy of the motor command and concurrent alpha-gamma motor neuron coactivation can provide further position-related information during active movements when an individual drives their limb’s muscles [8, 16–26]. Finally, forearm location may be communicated cross-hemispherically so that the position of the two arms can be compared [27].

In addition to determining whether individuals with stroke who have deficits on a between-arms task also have deficits on a single-arm task, we were also interested in whether the arm referenced affects participant position-matching (single arm) and position-mirroring (between arms) ability. While controversial, numerous bodies of work suggest that position-matching and position-mirroring ability depends on whether an individual references their dominant versus non-dominant arm [26, 28–38]. Furthermore, prior work underscores that the paretic arm and non-paretic arm each may have sensory impairments in individuals post-stroke [39–42]. Moreover, Hirayama et al. indicated that impairments on a between-arms task may depend on the arm referenced [6]. Specifically, they found that patients with a unilateral brain lesion were impaired when locating their paretic thumb, yet unimpaired when locating their non-paretic thumb. Therefore, we assessed participant position-matching ability in each arm, separately, and position-mirroring ability between-arms, bi-directionally (i.e., paretic forearm mirrors non-paretic forearm, non-paretic forearm mirrors paretic forearm).

In this manuscript, our goal was to determine whether individuals with chronic hemiparetic stroke had deficits when matching forearm positions within a single arm and mirroring forearm positions between arms, independent of the forearm referenced. We hypothesized, based on findings in [4] and [6], that participants with chronic hemiparetic stroke who were classified as having a position-mirroring deficit, yet an intact ability to detect the direction of their arm’s movement, would: i) not have a deficit when matching forearm positions within their paretic arm and non-paretic arm, separately (single-arm experiment), and ii) have a deficit when mirroring forearm positions between arms when their non-paretic forearm mirrored their paretic forearm, yet not have a deficit when their paretic forearm mirrored their non-paretic forearm. These findings would support the notion that employing a variety of single-arm and between-arms tasks provides a more complete assessment of an individual’s ability to locate their arm(s) in space than employing only one of these tasks, which currently is the approach often taken, e.g., [5, 43–46].

## Materials and methods

In designing our single-arm position-matching task and between-arms position-mirroring task, we were inspired by the clinical revised Nottingham Sensory Assessment (rNSA) elbow kinaesthesia test [3]. A gold standard does not currently exist to assess position sense [5, 43]. However, the Academy of Neurologic Physical Therapy StrokEDGE Taskforce underscored the improved clinical utility of the rNSA when compared to other sensory assessments [47], largely because of the kinaesthesia test. For the rNSA elbow kinaesthesia test, a clinician rotates the examined individual’s paretic forearm to a reference target location. Then, the examined individual rotates their non-paretic forearm, without sight, to mirror the location of their paretic forearm. The rNSA defines intact position sense as the ability to mirror forearm positions with a magnitude of positional error, between-arms, that is less than 10° and impaired position sense as a magnitude of positional error, between-arms, that is greater than 10°. Accurate direction of movement sense is defined as an ability to detect the direction of one’s forearm’s movements, whereas impaired direction of movement sense is defined as an inability to detect the direction of one’s forearm’s movements. Here, we adopted the clinical rNSA elbow kinaesthesia test to initially assess and screen participants using their classified position and direction of movement sense. Following, we used our robotic assessments, as inspired by this clinical rNSA test, to quantify position-matching ability and position-mirroring ability within an arm and between arms, respectively, when an individual’s reference arm is passively rotated and the matching/mirroring arm is actively controlled. A robotic system and automated protocol were used to quantify every participant’s ability to match and mirror forearm positions within each arm and between arms, respectively. A discussion of the tested participants, setup, and data collection protocol for each experiment is provided below and is presented in more detail in [4] and [48].

### Participants

The Northwestern University Institutional Review Board granted approval to run the single-arm position-matching and between-arms position-mirroring experiments (STU00021840). Eighteen individuals with chronic hemiparetic stroke and nine individuals without neurological impairments (i.e., controls) provided written informed consent to participate in each experiment. Non-University employees were monetarily compensated for their time.

Inclusion criteria for all participants included an ability to understand and perform the task; absence of serious upper-extremity injury, peripheral nerve injury, and antispastic pharmacological agents that may impact task performance; ability to actively control arm movements at <10°/s, which was needed to successfully complete the position-matching and position-mirroring tasks [48]; intact direction of movement sense; and capacity to provide informed consent, which was determined based on an individual’s ability to respond to questions and engage in conversation during the recruitment process, followed by a screening with a licensed physical therapist (co-author Dr. Justin Drogos, PT/DPT/NCS).

Demographic and clinical information for the participants with stroke are provided in Table 1. These participants were required to have a unilateral brain lesion at least one year prior. Their motor and sensory impairments were clinically assessed by the licensed physical therapist using the upper-extremity Fugl-Meyer Motor Assessment [49] and rNSA [3], respectively. Also, the licensed physical therapist screened participants for impairments that would exclude them from eligibility in this study. The licensed physical therapist identified that none of the participants had neglect, whereas sensory extinction at the elbow could not be excluded. Lesion locations were identified from medical records and T1/T2 MRI scans.

**Table 1:** Information relevant to participants with stroke. Provided are demographic and clinical information for each participant with stroke. Additionally, provided is the minimum and maximum active range-of-motion for the participant’s paretic arm, along with the minimum and maximum angles at which the participant actively matched and mirrored the reference target location using their paretic arm. Using this information, we verified that the ability of our participants with stroke to match and mirror positions was not compromised due to a limited active range-of-motion.

Participants with Stroke	rNSA Elbow Position Sense	Gender	Age	Dominant/Paretic Arm	Years since Stroke	Upper-Extremity FMA Score	Lesion Location(s) (R: Right, L: Left)	Min/Max Active Range-of-Motion of Paretic Arm	Min/Max Single-Arm Position-Matching Angle for Paretic Arm	Min/Max Between-Arms Position-Mirroring Angle for Paretic Arm
Stroke 1	Intact	F	67	R/R	12	11	L: Th, IC, BG	57.4°/126.0°	68.6°/115.0°	69.2°/109.2°
Stroke 2	Intact	M	61	L/R	12	50	L: IC	50.7°/181.6°	75.9°/108.8°	74.0°/111.0°
Stroke 3	Intact	F	61	R/L	3	28	R: IC, BG	55.6°/165.5°	73.2°/105.1°	73.1°/105.1°
Stroke 4	Intact	M	71	L/R	5	32	L: Po	62.6°/154.8°	70.8°/111.1°	71.9°/109.3°
Stroke 5	Intact	M	42	R/R	3	39	L: BG, T, F, P	57.1°/150.7°	72.0°/113.1°	78.8°/98.4°
Stroke 6	Intact	F	69	R/L	16	28	NA	59.3°/136.9°	^1^75.2°/102.7°	71.1°/108.5°
Stroke 7	Intact	M	63	R/R	17	59	NA	57.5°/157.0°	67.9°/112.2°	69.6°/127.6°
Stroke 8	Intact	M	61	L/L	6	17	R: IC, Po	61.5°/124.5°	76.1°/101.1°	76.1°/106.6°
Stroke 9	Impaired	M	46	R/L	11	35	R: Th, IC	46.7°/151.1°	72.8°/110.4°	77.4°/103.9°
Stroke 10	Impaired	M	69	L/R	21	12	L: Th, IC, BG, I	69.3°/128.3°	^1^^,^^2^94.1°/97.1°	^2^93.0°/99.4°
Stroke 11	Impaired	F	75	R/R	12	56	L: F	57.1°/152.1°	70.7°/115.0°	74.8°/122.0°
Stroke 12	Impaired	F	46	R/L	9	28	R: IC, SF, FP, T	66.0°/186.5°	78.2°/109.1°	76.1°/112.8°
Stroke 13	Impaired	M	50	R/L	17	30	R: Th, IC, BG, PO	^3^NA/NA	70.3°/110.6°	71.1°/126.4°
Stroke 14	Impaired	M	60	R/L	4	20	R: BG, F	63.4°/150.7°	75.0°/111.5°	92.4°/109.1°
Stroke 15	Impaired	M	59	R/L	9	25	R: Th, IC, BG	62.1°/107.1°	^4^73.9°/85.6°	^4^70.3°/76.8°
Stroke 16	Impaired	M	49	L/R	27	26	NA	51.0°/166.0°	71.0°/118.4°	66.6°/112.1°
Stroke 17	Impaired	F	62	R/R	29	13	L: Th, IC, BG	67.4°/129.8°	81.7°/104.8°	83.0°/101.4°
Stroke 18	Impaired	M	61	R/L	8	15	R: IC, BG	46.4°/102.9°	^1^^,^^4^81.0°/86.3°	^4^69.4°/72.6°

FMA: Fugl-Meyer Motor Assessment, NA: information not available, Th: thalamus, IC: internal capsule, BG: basal ganglia, F: frontal lobe, FP: frontal/parietal lobes, PO: parietal/occipital lobes; I: insula, T: temporal lobe, P: parietal lobe, SF: sylvian fissure, Po: pons, EC: external capsule, CR: coronoradiata.

^1^The flexion angle tested was 85°, not 77.5°. This adjusted angle was selected to ensure that the participant could, when matching, safely interact with the robotic device despite their limited range-of-motion and the positioning of their arm in the device with respect to the mechanical safety checks.

^2^Flexion trials when the non-paretic forearm was referenced were not analyzed due to a limited range-of-motion in the paretic arm.

^3^This participant had an active range-of-motion of 114.3° (i.e., max-min angle).

^4^Extension trials when the non-paretic forearm was referenced were not analyzed due to a limited range-of-motion in the paretic arm.

For the single-arm experiment, the nine controls were all right-hand dominant and had a median age of 59 (lower/upper quartile: 57/62; range: 49-66). They were comprised of five females and four males. For the between-arms experiment, we reference the controls’ data in [48]. The study included nine controls who were comprised of seven females and two males, were all right-hand dominant, and had a median age of 61 (lower/upper quartile: 57/62; range: 46-66). Five of the controls participated in each experiment, whereas the remaining controls participated in only the single-arm experiment or only the between-arms experiment. The control groups tested were not identical due to the consecutive nature of the experimental testing. Therefore, conclusions drawn by comparing their results on the single-arm and between-arms tasks are not as strong as had the same sample population of controls been tested in each experiment.

### Experimental setup

**Fig 1:**
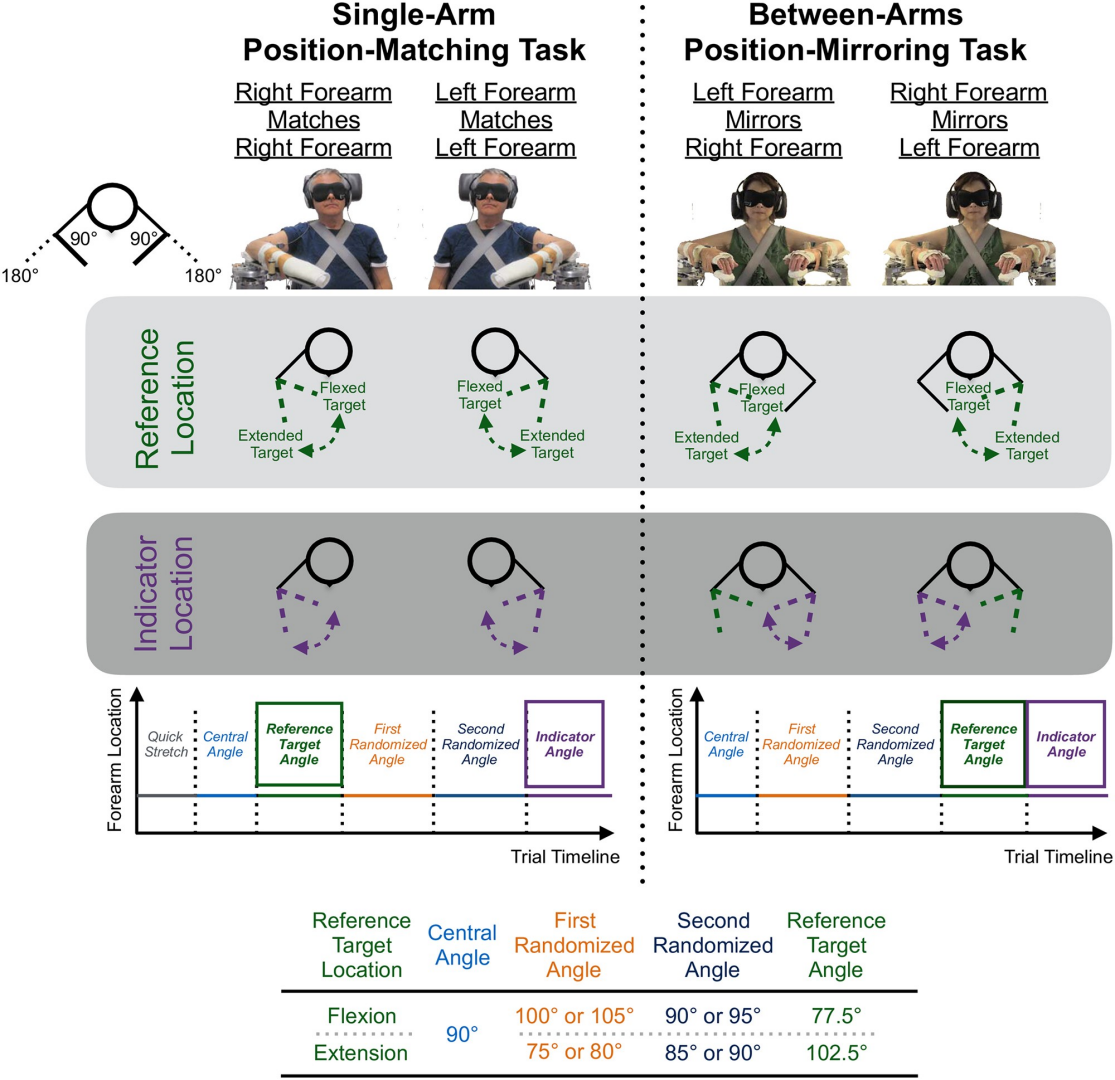
Experimental methods. Participants performed a (Left) single-arm position-matching task when using their right arm and their left arm and (Right) between-arms position-mirroring task when referencing their right arm and their left arm. The participant’s goal was to identify a reference target location with their reference forearm and then to return to that location with their same or opposite forearm. Angles to which the participant’s forearm rotated for each reference target location are indicated in the table at the bottom of the figure. The individuals shown in this figure provided written informed consent, as outlined in the PLOS consent form, to publish their photos. Images are adapted from Gurari et al., 2018 [48].

The experimental setups for the single-arm and between-arms experiments are shown in Fig 1. For each experiment, the participant’s reference forearm was attached to a custom robotic device. For the between-arms experiment only, the indicator forearm was attached to a custom passive measurement device. The robotic device included a Harmonic Drive FHA-17C-100 motor with an attached US250 encoder (Peabody, MA, USA), and the passive measurement device included a US Digital MA3 Miniature Absolute Magnetic Shaft Encoder (Vancouver, WA, USA). Movements of the robotic device and passive measurement device were controlled by the motor and by the participant and experimenter, respectively. As demonstrated in [48], the torque response of the robotic device and passive measurement device was comparable for the interaction velocities tested (i.e., <10°/s). Additional information about the robotic device and passive measurement device can be found in [4] and [48].

### Trial

For every trial, the participant’s reference forearm passively rotated about the elbow joint to one of two reference target locations (i.e., flexion, extension), and then the participant actively matched their reference forearm, when located at the reference target location, using their same or opposite forearm (i.e., indicator forearm) (see Fig 1). This sequence of events was designed to be the same sequence of events as the clinical revised Nottingham Sensory elbow kinaesthesia test [3]. We chose to quantify participant position-matching and position-mirroring ability, respectively, at a flexed and an extended location since position-localization ability can differ depending on the arm’s location. Prior work indicates that flexed locations are more accurately identified than extended locations for reasons including that the geometric properties are more ideal [50, 51] and overestimation occurs at an extended location to prevent injury due to over-stretching [19]. We propose that physical constraints arising due to a contracture in individuals with stroke [52] could also potentially impact position-mirroring ability, depending on the limb’s location. Therefore, position-matching and position-mirroring ability were assessed at two locations.

For each experiment, the participant’s forearm rotational speed was restricted to <10°/s to encourage comparable interaction speeds, and, in turn, comparable device torque responses [53]. The participant wore a blindfold and noise-canceling headphones to prevent visual and auditory cues from aiding their judgment. Moreover, the participant’s reference forearm was rotated to two random locations, in addition to the reference target location, to avoid the possibility that the reference target location could be determined based on timing cues.

The single-arm trials were executed first, using procedures similar to those presented in [4], and quantified participant forearm position-matching ability within each arm, separately. Mimicking the single-arm testing procedures of [4], the participant’s forearm initially was quickly rotated about the elbow joint to avoid effects of muscle thixotropy on position-matching ability [12, 14, 15]. Next, the between-arms trials were executed, as presented in [48], to quantify participant forearm position-mirroring ability. The quick stretch was not included for the between-arms task given our goal of quantifying participant task performance on the rNSA elbow kinaesthesia test [3].

Below we identify the events occurring within a trial for each experiment.

#### Single-arm trial

The participant’s testing forearm was passively rotated to the central angle (i.e., 90°), quickly stretched to avoid effects of muscle thixotropy [12, 14, 15], rotated to the reference target location, held still for 4 s, and then rotated to two randomized locations. Last, the participant actively rotated their same forearm to match the perceived reference target location held in working memory and verbally indicated when the forearm position was matched.

#### Between-arms trial

The events within a between-arms trial are described in [48] from testing conducted with the controls. Here, we describe the events for the reader. Both of the participant’s forearms were passively moved to the central angle (i.e., 90°). Next, the participant’s reference forearm was passively rotated to two randomized locations, rotated to the reference target location, and then held still for 4 s. Last, the participant actively rotated their opposite forearm to mirror the location of their reference forearm and verbally indicated when the forearm positions were mirrored.

### Procedures

To begin each session, the participant sat with their trunk and waist strapped to a Biodex chair (Shirley, NY, USA). The participant’s testing forearm(s) was outfitted with a fiberglass cast (Techform Premium 204WH; Reykjavik, Capital Region, Iceland). The cast encouraged a rigid connection between the participant’s forearm and the instrumented device, promoted participant comfort, and ensured that haptic cues transmitted across the length of the participant’s forearm. The weight of the participant’s arm(s) was fully supported by the device(s) to maximize the active range-of-motion in the paretic arm of participants with stroke by eliminating shoulder abduction induced limitations in elbow extension [54]; ensure that participant forearm mirroring ability was not affected by the weight of the arm [9, 38, 55]; and reduce the possibility of fatigue.

Next, the participant’s active range-of-motion about each elbow joint was determined by having the participant reach to maximal and minimal locations. This information made it possible for us to confirm that a participant with stroke’s motor impairments did not interfere with their ability to execute our position-matching and position-mirroring tasks.

#### Single-arm procedures

The single-arm experiment was run across two sessions. The chair was adjusted such that the participant’s testing arm had an anatomical shoulder angle of 90° abduction and 45° flexion, as employed in our previous single-arm study [4]. During each session, the participant completed a total of sixteen trials (eight flexion and eight extension reference target locations) for one of the arms, taking a minimum one minute break after eight trials. Presentation order of the trials was randomized within each session, and presentation order of the testing arm was randomized between sessions.

#### Between-arms procedures

Procedures for the between-arms experiment are described in [48] from testing conducted with the controls. Here, we briefly describe the procedures for the reader. The between-arms experiment was run across two sessions. The chair was adjusted such that the participant’s testing arms had anatomical shoulder angles of 85° abduction and 35° flexion. The shoulder angles for this between-arms task were slightly adjusted from the shoulder angles for the single-arm task to avoid the possibility that the participant’s fingers on each hand would touch during elbow flexion movements and to avoid participant discomfort from having both arms elevated for a long period of time. The participant completed a total of eight trials (four flexion and four extension reference target locations). Presentation order of the trials was randomized within each session, and presentation order of the arm referenced was randomized between sessions.

As indicated above, during the single-arm procedures the participant completed eight trials at the flexion location and eight trials at the extension location. This number of trials was identified in our earlier work as appropriate to characterize an individual’s position-matching ability [4]. For the between-arms procedures, the participant completed four trials at the flexion location and four trials at the extension location. An analysis discussed in [48] indicated that position-mirroring ability, as determined based on four trials, did not give significantly different results than position-mirroring ability, as determined based on eight trials.

## Data analyses

Below we discuss the methods used to analyze the data for each participant and experiment (i.e., single-arm, between-arms). Independent variables were reference arm (i.e., dominant, non-dominant in controls; paretic, non-paretic in participants with stroke), reference target location (i.e., flexion, extension), and group classification (i.e., according to our quantitative classification, as discussed below: Controls, participants with stroke who have an intact position-matching/-mirroring ability, or Participants with Stroke: Intact, and participants with stroke who have a position-matching/-mirroring deficit, or Participants with Stroke: Deficit). Data for the controls on the between-arms task were pulled from [48] so that we could compare the position-mirroring ability of our participants with stroke to a population without neurological impairments.

### Position-matching and position-mirroring abilities

Position-matching ability on the single-arm task and position-mirroring ability on the between-arms task were characterized for each participant, experiment, reference arm, and reference target location using the dependent measures of constant error, absolute error, and variable error [48, 56, 57]. First, we identified for each trial, *i*, the position-matching/-mirroring error, e_*i*_, or the difference between the matched/mirrored target location, m_*i*_, and reference target location, r_*i*_. Position-matching/-mirroring errors were defined such that positive and negative errors indicated that the participant overshot and undershot the reference target location, respectively. Position-matching/-mirroring errors were visually inspected as a function of trial to verify that learning, fatigue, or boredom, as would be indicated by trends of increasing or decreasing position-matching/-mirroring errors, did not occur.

Next, the dependent measures of constant error, absolute error, and variable error were obtained for each participant, experiment, reference arm, and reference target location. Constant error, $\text{CE}=\frac{\sum_{i=1}^{n}e_{i}}{n}$, is the mean position-matching/-mirroring error and identifies whether the participant tended to overshoot (CE > 0), undershoot (CE < 0), or accurately match/mirror the reference target location (CE = 0). Absolute error, $\text{AE}=\frac{\sum_{i=1}^{n}\left|e_{i}\right|}{n}$, is the mean absolute position-matching/-mirroring error and identifies the magnitude of the participant’s position-matching/-mirroring errors. Perfect matching/mirroring corresponds to AE = 0 and poor matching/mirroring corresponds to AE > > 0. Variable error, $\text{VE}=\sqrt{\frac{\sum_{i=1}^{n}{\left(e_{i}-\text{CE}\right)}^{2}}{n-1}}$, is the variance of the position-matching/-mirroring errors and identifies whether the participant consistently matched/mirrored at the same location (VE = 0) or was inconsistent in their matching/mirroring location (VE > 0).

### Evaluation of position-matching and position-mirroring deficits

We evaluated whether each participant had a position-matching deficit on the single-arm task and a position-mirroring deficit on the between-arms task using an analytical approach that was inspired by the revised Nottingham Sensory Assessment elbow kinaesthesia test. The rNSA test defines intact position sense as the ability to mirror forearm positions with a magnitude of positional error, between-arms, that is less than 10° and impaired position sense as a magnitude of positional error, between-arms, that is greater than 10°. The rNSA test assesses individuals at both flexed and extended locations. Inspired by this test, and using our quantitative measures, we defined a position-matching deficit and a position-mirroring deficit based on the magnitude of positional error, or the absolute error, from each arm of the controls in flexion and extension.

First, we identified for the single-arm robotic assessment the cutoff angle of a position-matching deficit. The position-matching deficit threshold angle was evaluated based on the controls’ absolute error in flexion and extension in their dominant arm and non-dominant arm (i.e., four conditions). We defined the position-matching deficit threshold angle as, across the four conditions in controls, the mean plus three standard deviations of the position-matching absolute error.

Next, we classified a participant with stroke as having a position-matching deficit if their flexion absolute error or their extension absolute error in either arm during the position-matching task (i.e., single-arm experiment) was greater than the position-matching deficit threshold. Otherwise, the participant with stroke was classified as having an intact ability to match forearm positions. Hence, we separated participants into three groups for the single-arm assessment: Controls, Participants with Stroke: Intact, and Participants with Stroke: Deficit.

Following, we identified for the between-arms robotic assessment the cutoff angle of a position-mirroring deficit. The position-mirroring deficit threshold angle was evaluated based on the controls’ absolute error in flexion and extension when referencing their dominant arm and non-dominant arm (i.e., four conditions). We defined the position-mirroring deficit threshold angle as, across the four conditions in controls, the mean plus three standard deviations of the position-mirroring absolute error.

Next, we classified a participant with stroke as having a position-mirroring deficit if their flexion absolute error or their extension absolute error when referencing either arm during the position-mirroring task (i.e., between-arms experiment) was greater than the position-mirroring deficit threshold. Otherwise, the participant with stroke was classified as having an intact ability to mirror forearm positions. Hence, we separated participants into three groups for the between-arms assessment: Controls, Participants with Stroke: Intact, and Participants with Stroke: Deficit.

#### Reference arm and reference target location

We investigated whether the independent factors of reference arm and reference target location significantly affected position-matching ability (i.e., single-arm experiment) and position-mirroring ability (i.e., between-arms experiment). First, for each classified group of participants of each experiment we determined whether the reference arm, reference target location, or their interaction significantly affected the dependent variables [58]. We fit the data to linear mixed-effects models with repeated measures [59]. Independent variables were treated as fixed effects, and participant was treated as a random effect. We identified which independent variables significantly affected which dependent variables, including interactions between the independent variable levels. The model was selected using a hierarchical approach in which non-significant interaction terms were removed followed by non-significant main effect terms. P-values were evaluated and significantly differing levels were determined using custom contrasts; p-values were adjusted using a Holm correction [60]. Last, estimated least-squares mean and standard error values were identified.

#### Group classification

We determined whether position-matching ability (i.e., single-arm experiment) and position-mirroring ability (i.e., between-arms experiment) at each reference target location significantly differed depending on a participant’s group classification. First, we determined for each experiment whether the dependent variables significantly differed for Controls when referencing their dominant arm, Participants with Stroke: Intact when referencing their paretic arm, and Participants with Stroke: Deficit when referencing their paretic arm [61]. Then, we determined for each experiment whether the dependent variables significantly differed for Controls when referencing their dominant arm, Participants with Stroke: Intact when referencing their non-paretic arm, and Participants with Stroke: Deficit when referencing their non-paretic arm. We fit the data to linear mixed-effects models [62]; group classification was treated as a fixed effect, and participant was treated as a random effect. The model was selected using a hierarchical approach in which non-significant interaction terms were removed followed by non-significant main effect terms. Significant effects were evaluated, and significantly differing levels were determined using pairwise comparisons [63]; p-values were adjusted using a Holm correction [60]. Following, estimated least-squares mean and standard error values were identified.

## Results

Below we present results for the single-arm experiment, followed by results for the between-arms experiment.

### Single-arm experiment

Prior to analyzing the single-arm position-matching data, we removed seven of the 864 trials since, based on visual inspection, it was clear that the participant moved to the incorrect remembered reference target location (e.g., flexion rather than extension). In addition, 24 trials were removed from the analyses because of the limited active range-of-motion of three participants with stroke; specifically, eight trials for Stroke 15 and 18 in extension and eight trials for Stroke 10 in flexion were removed. Data from the remaining 833 trials were analyzed.

Based on the controls’ absolute error data, we identified a position-matching deficit threshold of 10.7°. That is, participants with stroke who had an absolute error greater than this threshold were classified as having a position-matching deficit. Using this threshold, only one participant with stroke, Stroke 16, was identified as having a deficit on the single-arm position-matching task.

**Fig 2:**
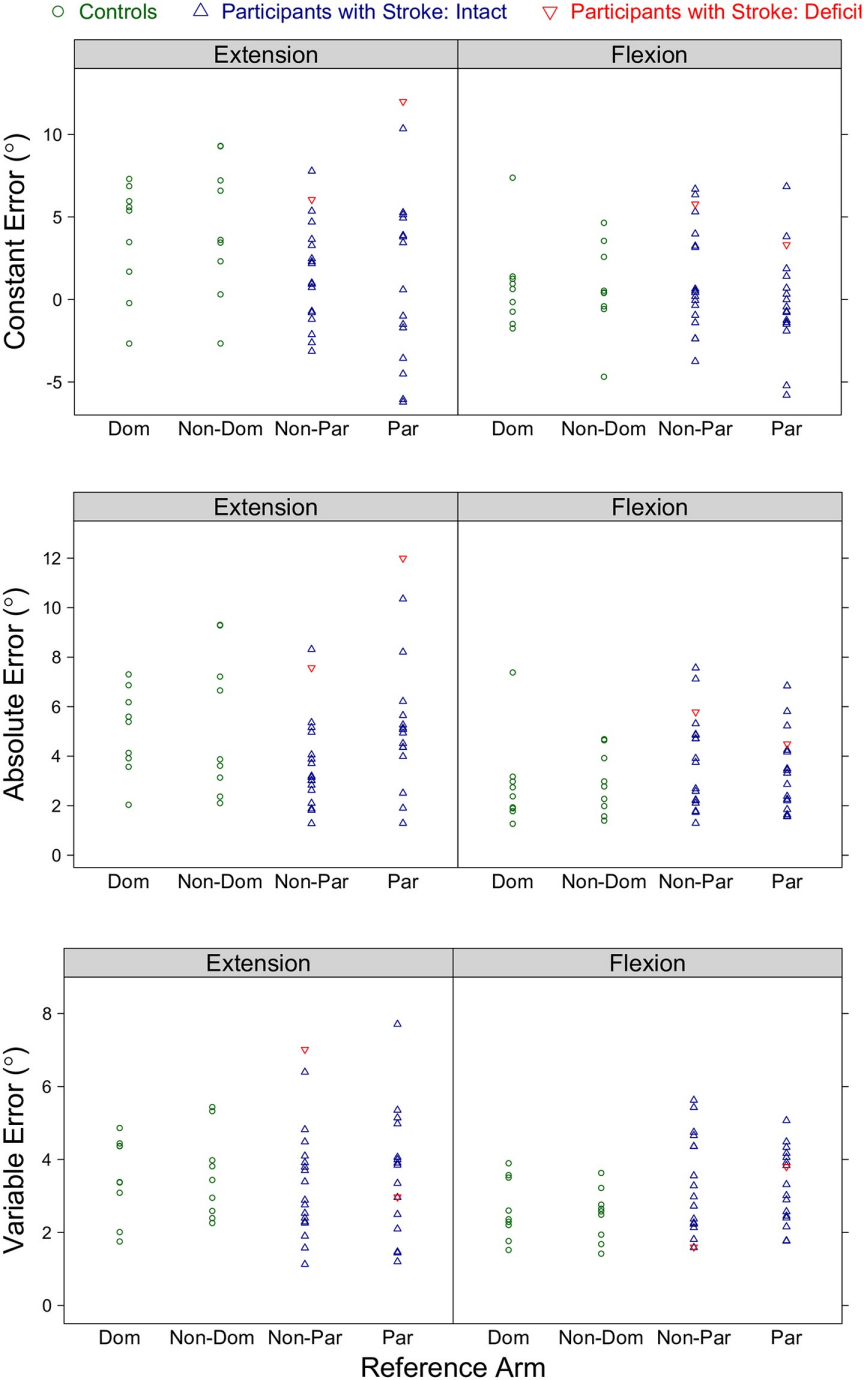
Single-arm position-matching results for each participant across all tested conditions. Shown are the robotic assessment results on the single-arm position-matching task for the controls and participants with stroke. Each participant’s (Top) constant error, (Middle) absolute error, and (Bottom) variable error are given as a function of the reference arm and reference target location. (Dom: dominant, Non-Dom: non-dominant, Non-Par: non-paretic, Par: paretic).

Results for the controls and participants with stroke across all tested conditions are given in Fig 2.

#### Position-matching ability as a function of reference arm and reference target location

**Fig 3:**
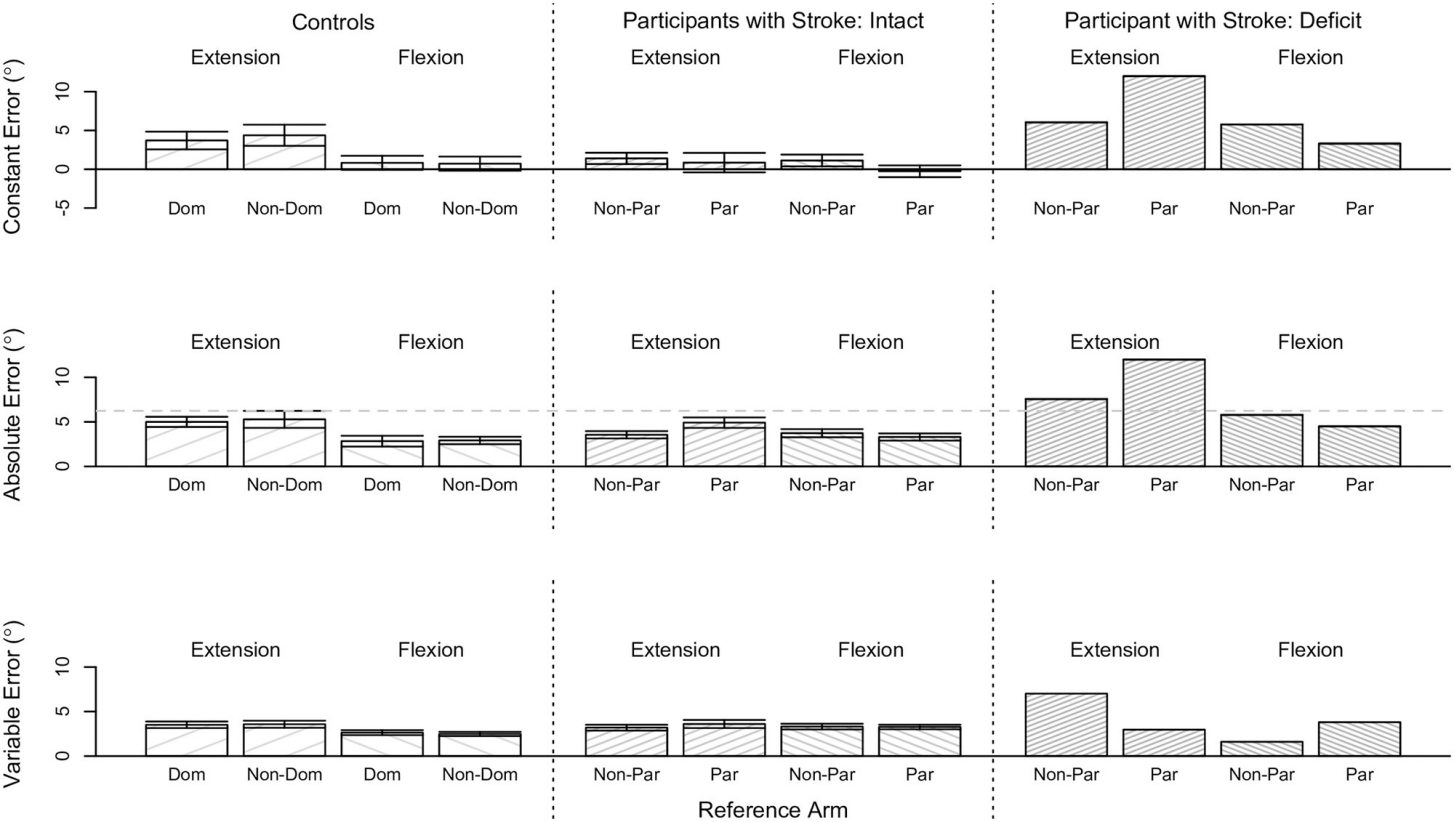
Single-arm position-matching results across all tested conditions. Shown are the robotic assessment results on the single-arm position-matching task for the controls and participants with stroke. Mean (bar height) and standard error (error bars) of the (Top) constant error, (Middle) absolute error, and (Bottom) variable error are given as a function of the reference arm and reference target location for each classified group of participants. The gray dashed horizontal line indicates the deficit threshold of 10.7°. No significant effects were found. (Dom: dominant, Non-Dom: non-dominant, Non-Par: non-paretic, Par: paretic).

We determined for each classified group of participants whether reference arm, reference target location, or their interaction significantly affected position-matching accuracy (i.e., CE, AE) and precision (i.e., VE). Results for the controls and participants with stroke across all tested conditions are summarized in Fig 3.

#### Controls

Reference arm, reference target location, and their interaction did not significantly affect the accuracy and precision of Controls when matching forearm positions (p>0.050).

#### Participants with stroke: Intact

Reference arm, reference target location, and their interaction did not significantly affect the accuracy and precision of Participants with Stroke: Intact when matching forearm positions (p>0.050).

#### Participant with stroke: Deficit

This analysis was not run since only one participant with stroke was classified as having a position-matching deficit.

#### Position-matching ability as a function of group classification

**Fig 4:**
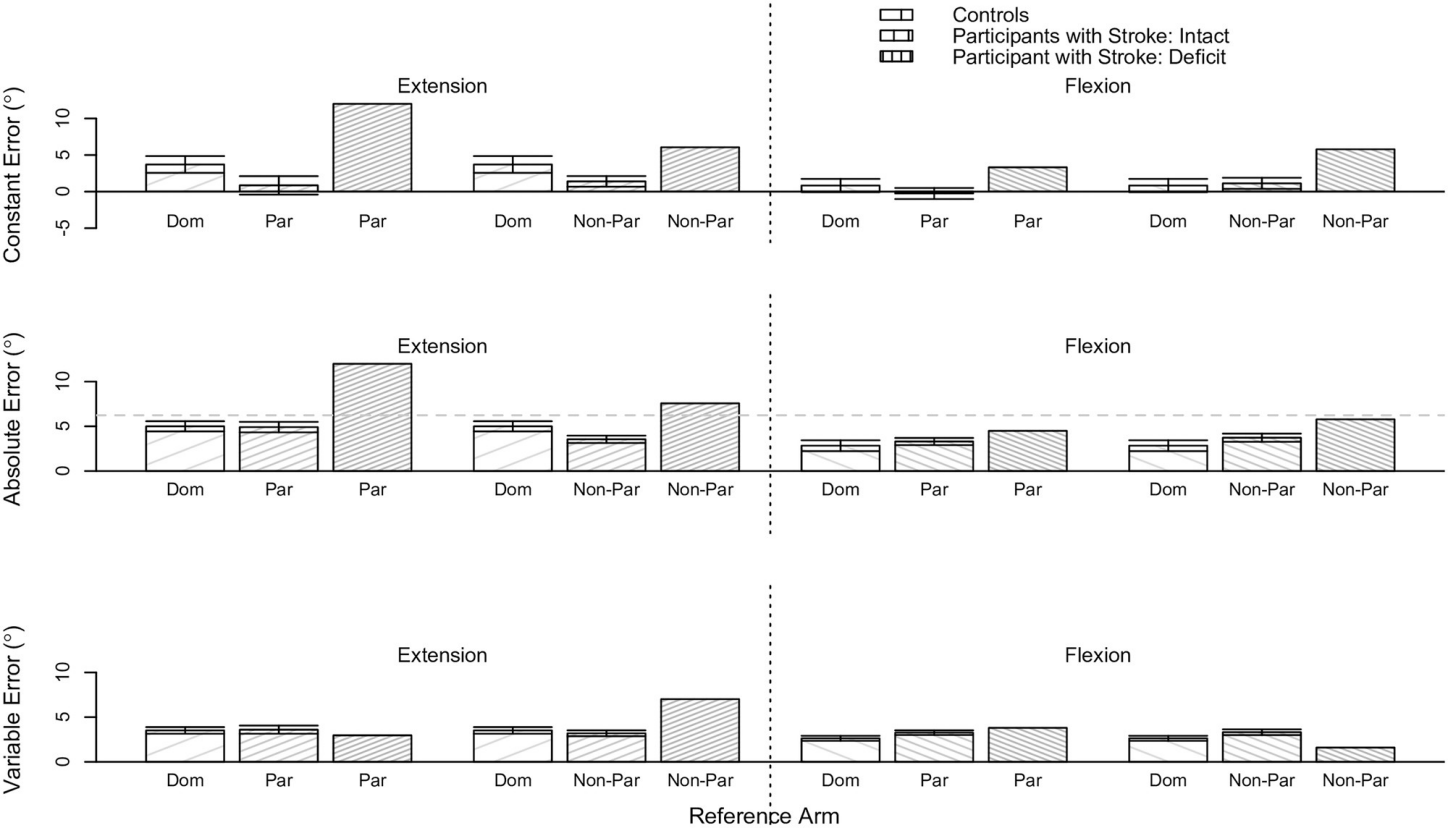
Single-arm position-matching results as a function of group classification. Shown are the robotic assessment results for the single-arm position-matching task based on our classification of participants. Mean (bar height) and standard error (error bars) of (Top) constant error, (Middle) absolute error, and (Bottom) variable error are given as a function of the reference arm and reference target location for each classified group of participants. The gray dashed horizontal line indicates the deficit threshold of 10.7°. No significant effects were found. (Dom: dominant, Non-Dom: non-dominant, Non-Par: non-paretic, Par: paretic).

We determined whether results on the single-arm position-matching task significantly differed depending on a participant’s group classification. We analyzed the data at the flexion and extension location, and for the non-dominant (controls) / paretic (participants with stroke) arm and dominant (controls) / non-paretic (participants with stroke) arm separately. Fig 4 provides the same data as Fig 3, yet visually portrays the results slightly differently to highlight the findings from the following analyses.

#### Extension location

Controls, Participants with Stroke: Intact, and Participant with Stroke: Deficit did not significantly differ in their constant error, absolute error, and variable error when referencing their dominant arm, paretic arm, and paretic arm, respectively (p>0.050); and their dominant arm, non-paretic arm, and non-paretic arm, respectively (p>0.050).

#### Flexion location

Controls, Participants with Stroke: Intact, and Participant with Stroke: Deficit did not significantly differ in their constant error, absolute error, and variable error when referencing their dominant arm, paretic arm, and paretic arm, respectively (p>0.050); and their dominant arm, non-paretic arm, and non-paretic arm, respectively (p>0.050).

### Between-arms experiment

Prior to analyzing the between-arms position-mirroring data, one of the 432 trials was removed since data were not recorded due to an experimental error. In addition, 24 trials were removed from the analyses because of the limited active range-of-motion of three participants with stroke; specifically, eight trials for Stroke 15 and 18 in extension and eight trials for Stroke 10 in flexion were removed. Data from the remaining 407 trials were analyzed.

Based on the controls’ absolute error data, we identified a position-mirroring deficit threshold of 10.1°. That is, participants with stroke who had an absolute error greater than this threshold were classified as having a position-mirroring deficit. Using this threshold, we identified seven participants with stroke, Stroke 6-7, 11-14, and 16, as having a deficit on the between-arms task. Results for the nine controls were previously presented and published in [48]. We include their data here so that the position-mirroring ability of our participants with stroke can be discussed in relation to the position-mirroring ability of individuals without neurological impairments.

**Fig 5:**
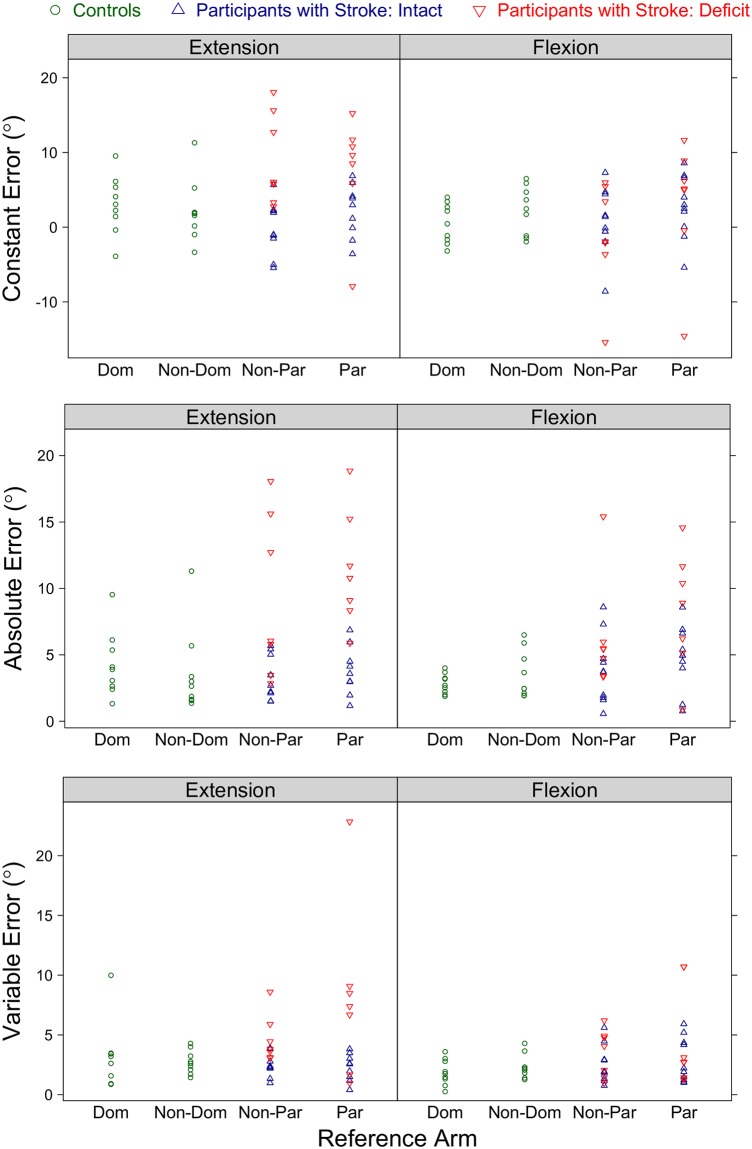
Between-arms position-mirroring results for each participant across all tested conditions. Shown are the robotic assessment results on the between-arms position-mirroring task for the controls and participants with stroke. Each participant’s (Top) constant error, (Middle) absolute error, and (Bottom) variable error are given as a function of the reference arm and reference target location. The controls’ data are reproduced from [48] to permit comparison of the between-arms position-mirroring ability of our participants with stroke to that of individuals without neurological impairments. (Dom: dominant, Non-Dom: non-dominant, Non-Par: non-paretic, Par: paretic).

Results for the controls and participants with stroke across all tested conditions are given in Fig 5.

#### Position-mirroring ability as a function of reference arm and reference target location

**Fig 6:**
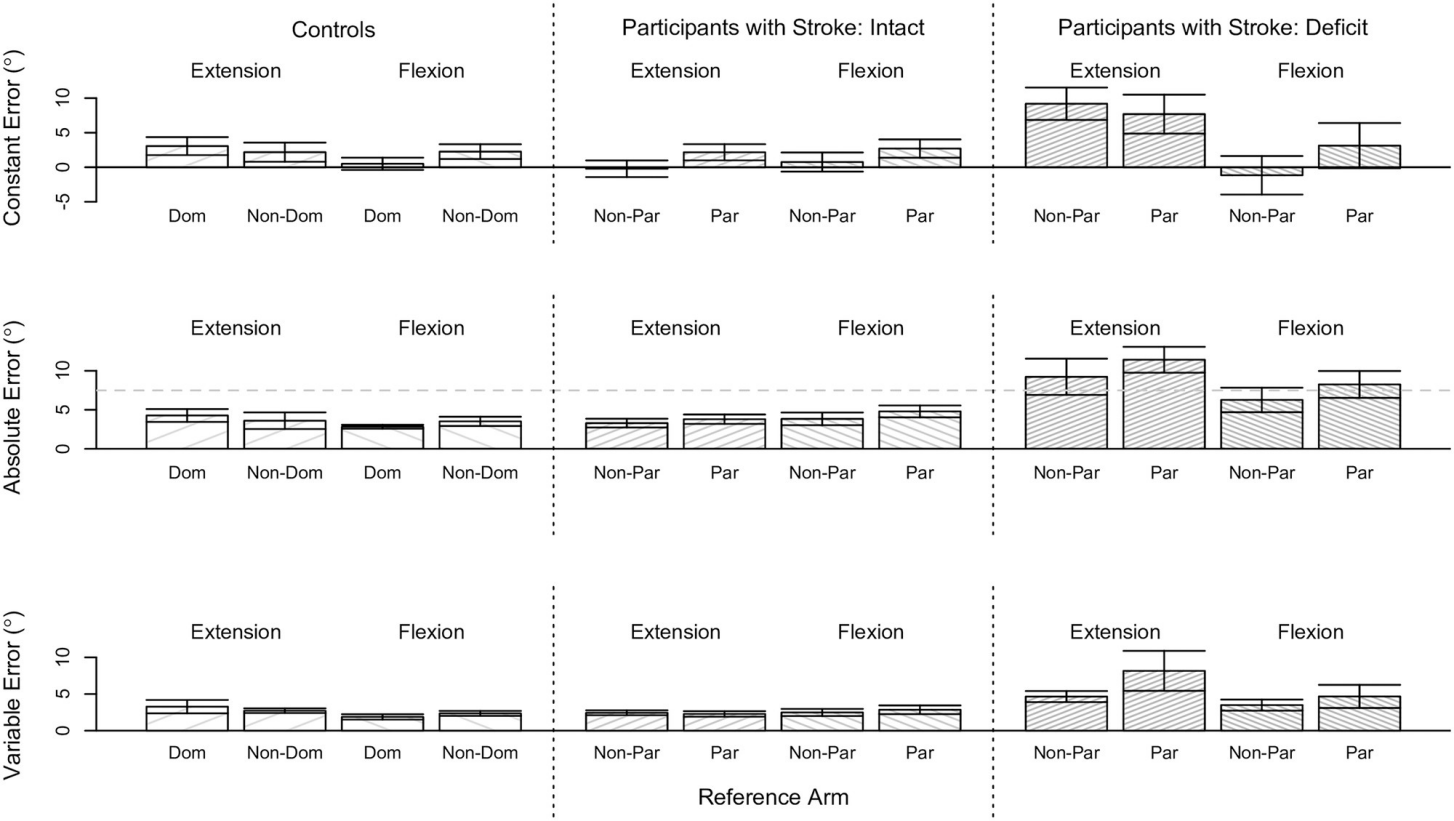
Between-arms position-mirroring results across all conditions. Shown are the robotic assessment results on the between-arms position-mirroring task for the controls and participants with stroke. Mean (bar height) and standard error (error bars) of the (Top) constant error, (Middle) absolute error, and (Bottom) variable error are given as a function of the reference arm and reference target location for each classified group of participants. The gray dashed horizontal line indicates the deficit threshold of 10.1°. No significant effects were found. The controls’ data are reproduced from [48] to permit comparison of the between-arms position-mirroring ability of our participants with stroke to that of individuals without neurological impairments. (Dom: dominant, Non-Dom: non-dominant, Non-Par: non-paretic, Par: paretic).

We determined for each classified group of participants whether the reference arm, reference target location, or their interaction significantly affected position-mirroring accuracy and precision. Results for the controls and participants with stroke across all tested conditions are summarized in Fig 6.

#### Controls

Reference arm, reference target location, and their interaction did not significantly affect the accuracy and precision of Controls when mirroring forearm positions (p>0.050). (Reproduced from [48]).

#### Participants with stroke: Intact

Reference arm, reference target location, and their interaction did not significantly affect the accuracy and precision of Participants with Stroke: Intact when mirroring forearm positions (p>0.050).

#### Participants with stroke: Deficit

Reference arm, reference target location, and their interaction did not significantly affect the accuracy and precision of Participants with Stroke: Deficit when mirroring forearm positions (p>0.050).

#### Position-mirroring ability as a function of group classification

**Fig 7:**
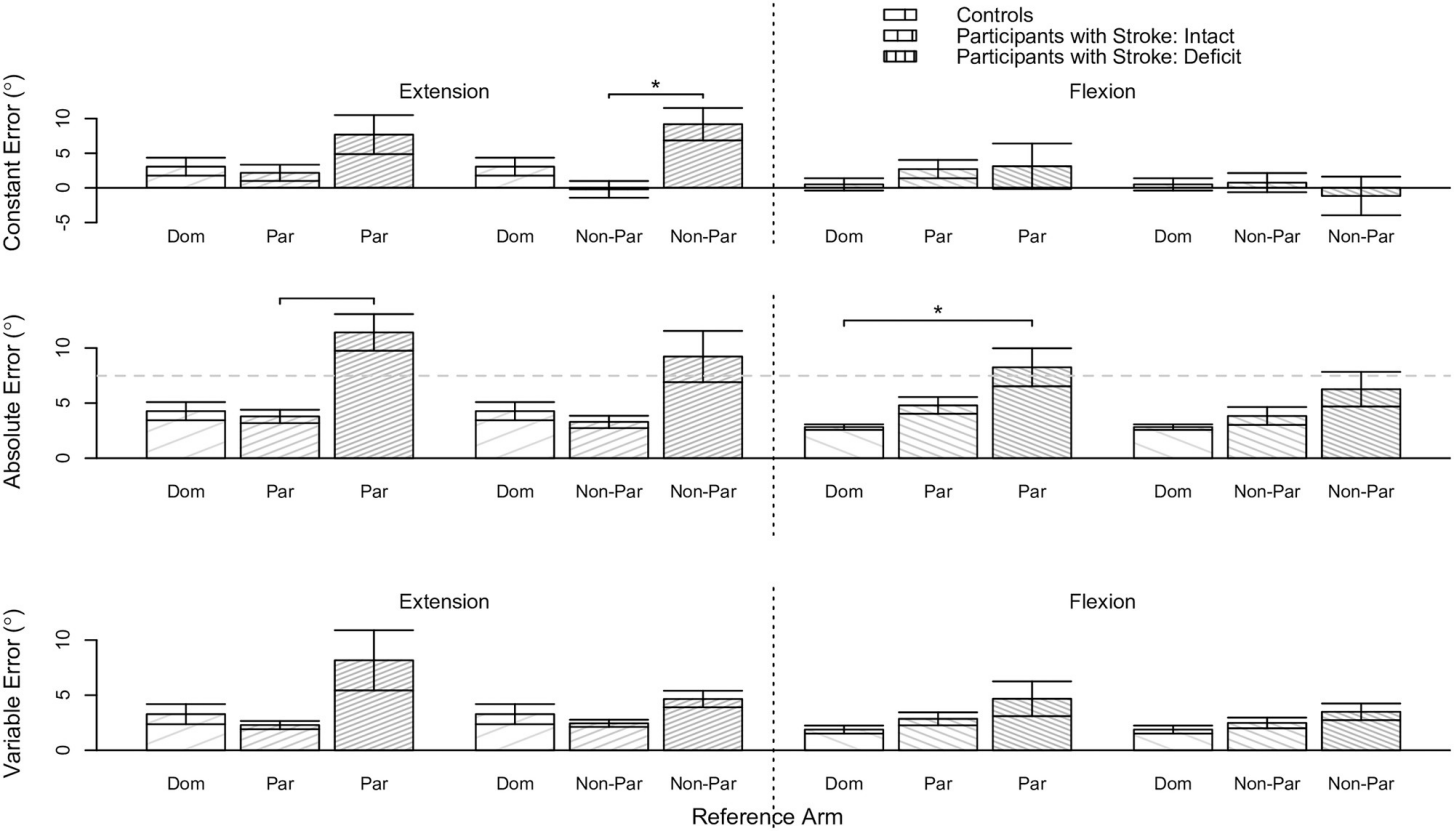
Between-arms position-mirroring results as a function of group classification. Shown are the robotic assessment results for the between-arms position-mirroring experiment based on our classification of participants. Mean (bar height) and standard error (error bars) of the (Top) constant error, (Middle) absolute error, and (Bottom) variable error are given as a function of the reference arm and reference target location for each classified group of participants. The gray dashed horizontal line indicates the deficit threshold of 10.1°. Black solid horizontal lines with a star above indicate significant effects. The controls’ data are reproduced from [48] to permit comparison of the between-arms position-mirroring ability of our participants with stroke to that of individuals without neurological impairments. (Dom: dominant, Non-Dom: non-dominant, Non-Par: non-paretic, Par: paretic).

We determined whether results on the between-arms position-mirroring task significantly differed depending on a participant’s group classification. We analyzed data at the flexion and extension location, and when referencing the non-dominant (controls) / paretic (participants with stroke) arm and dominant (controls) / non-paretic (participants with stroke) arm separately. Fig 7 provides the same data as Fig 6, yet visually portrays the results slightly differently to highlight the findings from the following analyses.

#### Extension location

The constant error significantly differed for Controls, Participants with Stroke: Intact, and Participants with Stroke: Deficit when referencing their dominant arm, non-paretic arm, and non-paretic arm, respectively (p = 0.006). The post-hoc analysis revealed that the constant error was significantly less for Participants with Stroke: Intact (estimated mean ± standard error: 3.29±1.23) than Participants with Stroke: Deficit (estimated mean ± standard error: 9.22°±1.39°) (p = 0.012); the estimated mean ± standard error of the constant error for Controls was 4.27°±1.23°.

The absolute error significantly differed for Controls, Participants with Stroke: Intact, and Participants with Stroke: Deficit when referencing their dominant arm, paretic arm, and paretic arm, respectively (p = 0.001). The post-hoc analysis revealed that the absolute error was significantly greater for Participants with Stroke: Deficit (estimated mean ± standard error: 11.41°±1.11°) than Controls (estimated mean ± standard error: 4.27±0.98) (p = 0.002) and Participants with Stroke: Intact (estimated mean ± standard error: 3.79±0.98) (p = 0.001). The absolute error also significantly differed for Controls, Participants with Stroke: Intact, and Participants with Stroke: Deficit when referencing their dominant arm, non-paretic arm, and non-paretic arm, respectively (p = 0.021). However, the post-hoc analysis did not reveal any significant effects between each group of participants (p>0.050).

#### Flexion location

The absolute error significantly differed for Controls, Participants with Stroke: Intact, and Participants with Stroke: Deficit when referencing their dominant arm, paretic arm, and paretic arm, respectively (p = 0.010). The post-hoc analysis revealed that the absolute error was significantly less for Controls (estimated mean ± standard error: 2.82±0.94) than Participants with Stroke: Deficit (estimated mean ± standard error: 8.25°±1.06°) (p = 0.021); the estimated mean ± standard error of the absolute error for Participants with Stroke: Intact was 4.79°±0.89°.

## Discussion

Our goal was to, using quantitative measures, determine whether individuals with chronic hemiparetic stroke had a deficit on a single-arm position-matching task and a between-arms position-mirroring task, independent of the arm that was referenced. This work was inspired by the limitations of our earlier work in which between-arms position-mirroring ability was not quantified and single-arm position-matching ability for a combination of passive and active movements was not identified [4]. The overarching aim of this research direction is to advance our understanding for the scenarios during which individuals with chronic hemiparetic stroke who have an intact perception of their limb’s direction of movement may have a compromised perception of their limb’s position. This research direction may lead to the design of more effective neurorehabilitative treatments, based on a more complete understanding of the extent of proprioceptive impairment(s), in individuals with neurological impairments, such as stroke.

The main findings of this work are as follows. First, individuals with chronic hemiparetic stroke who were classified as having a position-mirroring deficit indeed had significantly greater between-arms position-mirroring errors, when referencing their paretic arm, than individuals who did not have neurological impairments. Hence, our results confirm that the between-arms task identifies a between-arms position-mirroring deficit. Second, results reveal that all but one of the participants with stroke who had a between-arms position-mirroring deficit did not have a single-arm position-matching deficit. Therefore, these results corroborate the findings of our earlier work indicating that an individual with stroke who has a between-arms position-mirroring deficit does not necessarily have a single-arm position-matching deficit [4]. In summary, our results demonstrate that assessments of single-arm position-matching ability and between-arms position-mirroring ability can lead to differing conclusions about the presence of a deficit. Furthermore, our results bring to question what is the reason that a deficit occurs on a between-arms task. Below we discuss the main findings for our single-arm and between-arms experiments in more detail.

### Position-matching/-mirroring ability

Position-matching ability and position-mirroring ability was similar in our controls during the single-arm conditions and between-arms conditions, respectively. Across all conditions, the mean constant error spanned 0.72° to 4.38° for the single-arm task and 0.50° to 3.06° for the between-arms task; the mean absolute error spanned 2.83° to 5.28° for the single-arm task and 2.82° to 4.27° for the between-arms task; and the mean variable error spanned 2.59° to 3.58° for the single-arm task and 1.88° to 3.28° for the between-arms task (see Figs 3 and 6). Moreover, the accuracy of our controls was comparable to the accuracy of tested adolescents [64] and adults [5, 32] without neurological impairments. Therefore, our results in controls corroborate the finding that position-matching accuracy about the elbow joint within a single arm is similar to position-mirroring accuracy about the elbow joint between arms [5, 32, 64].

Based on the controls’ data, we obtained a position-matching deficit threshold of 10.7° and a position-mirroring deficit threshold of 10.1°. These deficit threshold angles are similar to the rNSA deficit threshold angle of 10.0°. Moreover, all of these deficit threshold angles are clinically similar, with less than a 0.7° difference, further supporting the notion that elbow position-matching accuracy (single arm) is similar to elbow position-mirroring accuracy (between arms) in individuals without neurological impairments.

Across all tested single-arm conditions, the mean magnitude of participant position-matching errors for each arm at each reference target location for seventeen of the eighteen participants with stroke did not exceed the 10.7° deficit threshold. Moreover, these seventeen participants with stroke did not significantly differ from the controls when matching forearm positions within their paretic arm and within their non-paretic arm.

Across all tested between-arms conditions, the mean magnitude of position-mirroring errors for each arm at each reference target location for seven of the eighteen participants with stroke exceeded the 10.1° deficit threshold. Six of these seven participants with stroke did not exceed the single-arm position-matching 10.7° deficit threshold and did not significantly differ from the controls on the single-arm task. Yet, these participants with stroke had significantly greater between-arms position-mirroring errors than controls when their non-paretic forearm mirrored their paretic forearm. Hence, these data demonstrate that results on a single-arm task and between-arms task can lead to differing conclusions about an individual’s awareness of the location of their limb(s) in space.

We underscore, based on our results, the point that a between-arms position-mirroring deficit may not be indicative of a single-arm position-matching deficit. Findings from Hirayama et al. indirectly addressed this idea by showing that 26.5% of 221 individuals with lesions were impaired on a between-arms thumb localization task, yet not on a single-digit direction of movement detection task [6]. Hence, future work needs to address the reason for a between-forearms position-mirroring deficit. A potential reason is that position information is accurate for the paretic arm and/or non-paretic arm, yet is inaccurately processed when compared.

Position-matching/-mirroring ability was not found to be significantly affected by whether the participant rotated their forearm to a flexed versus an extended location. Significant differences may have been found if flexion and extension locations had been tested that were more extreme and if data were collected from additional participants. Yet, the differences likely would not have been great enough for distinction when using a goniometer to measure elbow joint angles in the clinical setting [65, 66].

### Control of motor impairments

We underscore that our findings summarize perceptual results and are not confounded by an individual’s motor impairments. All participants with stroke were screened by a licensed physical therapist for potentially relevant motor impairments (e.g., apraxia, dysmetria). In addition, we verified that participant position-matching/-mirroring ability was not affected by a limited active range-of-motion (see Table 1), which could occur for reasons including weakness and contractures. Finally, we controlled for the abnormal joint coupling that can occur in individuals with stroke by supporting the weight of the paretic arm [54]. Therefore, despite the fact that most of our participants with stroke had moderate to severe motor impairments, we confirmed that their ability to execute the perceptual task was not compromised.

### Limitations of study

Below we highlight limitations when interpreting the results of this work.

One limitation of this study is that we classified participants and analyzed their data based on their absolute error. A challenge faced in this research area is determining how to appropriately assess a deficit. That is, should the assessment include single-arm and/or between-arms tasks?; testing at a flexion and/or extension location?; passive and/or active movements? Moreover, which criteria should be used to identify a deficit (e.g., CE, AE, VE)? Given the limited understanding currently available of the reason for a position-localization deficit, we underscore that future research needs to investigate the reason for the deficit so that an appropriate assessment approach and outcome measure can be employed. We adopted our approach since it resembles the approach of the clinical rNSA elbow kinaesthesia test in that a deficit is classified based on the magnitude of the error at flexion and extension locations for the individual tested [3]. Instead of classifying deficits based on a participant’s accuracy, as we had done, we could have classified deficits based on a participant’s precision. Had we classified participants as having a deficit based on their variable error, rather than their absolute error, Stroke 12, 13, and 16 would have had a deficit on the single-arm task and Stroke 12-14 and 16 on the between-arms task. Therefore, the position-matching deficit would have corresponded better with a position-mirroring deficit than was observed based on the absolute error outcome measure.

A second limitation is that the methods for the between-arms task did not exactly mimic the methods for the single-arm task. Differences exist because the robotic assessment for the latter was designed and tested only after the robotic assessment for the former was completed. Consequently, the anatomical location of the elbow and shoulder joints for the between-arms task were slightly modified from the anatomical configuration for the single-arm task to ensure that a participant’s fingers would not touch and to avoid participant pain/fatigue. In turn, the biceps may have been shorter for the between-arms task than the single-arm task, leading to possible differences in findings for each task. An additional potential cause for differences in results is that the single-arm task included a quick stretch prior to each trial to avoid effects of muscle thixotropy [12, 14, 15]. This quick stretch was not included in the between-arms task since our aim was to quantify participant performance on the rNSA elbow kinaesthesia test, which does not include the quick stretch. Finally, the number of testing trials at each reference target location was eight for the single-arm task and four for the between-arms task. We acknowledge that our results would have been strengthened had the same number of trials been tested for each task. Even so, an analysis presented in [48] demonstrated that results on between-arms position-mirroring tasks did not significantly differ when the analysis was run on eight trials versus four trials.

We underscore that the findings presented here are relevant to individuals with chronic hemiparetic stroke who have, according to the rNSA elbow kinaesthesia test, an intact direction of movement sense. During our testing for the experiments in [4], we discovered that the two individuals with stroke who were clinically assessed as having an impaired direction of movement sense were unable to match to passively-imposed positions.

We also highlight that, due to the consecutive nature of the experimental testing, the controls who participated in the single-arm experiment and between-arms experiment were not identical. However, five of the nine controls participated in both experiments, giving some continuity to the control population. The experimental results would have been further strengthened had the control population been exactly the same for each experiment.

Finally, we emphasize that the results were for individuals with hemiparetic stroke who were often very chronic, ranging from three to 29 years post stroke. Hence, our findings may not extend to those in the acute and sub-acute phases of stroke when neuroplasticity is more likely to occur.

### Clinical relevance

Our results support the validity of the clinical rNSA elbow kinaesthesia test [3] for identifying a between-arms position-mirroring deficit. The threshold angle for identifying a deficit is similar for our study and for the rNSA test, where the between-arms deficit threshold for our study was 10.1° and for the rNSA test was 10°. Even though the magnitude of error defining a deficit was similar for both approaches, the classification of participants as having a position-mirroring deficit was not identical. Four participants with stroke who were classified as having a position-mirroring impairment on the rNSA elbow kinaesthesia test were classified as having an intact position-mirroring ability based on our quantitative approach, and two participants with stroke who were classified as having an intact position-mirroring ability on the rNSA elbow kinaesthesia test were classified as having a position-mirroring deficit based on our quantitative approach. Differences in the classification of participants arose for reasons including that: i) the clinical assessment relies on an experimenter’s subjective evaluation (i.e., visual observation) whereas our approach relies on a quantitative objective evaluation (i.e., data from a position sensor), and ii) the clinical assessment classifies a deficit based on data arising from a single trial whereas our approach classifies a deficit based on data arising from all tested trials.

Given our findings here and in [4], we indicate that a need exists for a battery of assessments that will account for participant position-localization ability on a range of tasks, including single-arm and between-arms tasks during various passively- and actively-controlled movements when referencing each arm. Robotic and clinical assessments currently exist to assess position and direction of movement sense in individuals with stroke. However, these assessments tend to assess participants on either a single-arm task or a between-arms task, and during passive only or a combination of passive and active movements [5, 43–46]. By incorporating a battery of assessments, a more comprehensive understanding of the degree to which position-localization deficits exist can be obtained.
